# Unilateral Right Thyrolinguofacial Trunk Arising From the External Carotid Artery Observed in an African Male Cadaver

**DOI:** 10.7759/cureus.81203

**Published:** 2025-03-25

**Authors:** John O Juma, Peter J Ward

**Affiliations:** 1 Human Anatomy, Kampala International University, Dar Es Salaam, TZA; 2 Biomedical Sciences, West Virginia School of Osteopathic Medicine, Lewisburg, USA

**Keywords:** external carotid artery, facial artery, lingual artery, linguofacial trunk, surgical anatomy, thyroid artery, thyrolinguofacial trunk

## Abstract

A common origin of the superior thyroid artery, lingual artery, and facial artery is an infrequent anatomic variation. This uniqueness could stem from an unusual regression of vessels during remodeling and angiogenesis. Here we present a case of thyrolinguofacial trunk that arose from the external carotid artery of a 47-year-old male cadaver encountered during routine dissection of the head and neck in the department of anatomy. The thyrolinguofacial trunk originated from the anterior surface of the right external carotid artery, from which it gave off the superior thyroid artery and a linguofacial trunk. The linguofacial trunk then gave off the lingual and facial arteries. Since surgery in this region often assumes the normal anatomical arrangement of structures, understanding both the normal and possible abnormal branching patterns of the common carotid artery could help prevent complications, such as hemorrhagic episodes, during operations on the face and neck.

## Introduction

Structures of the neck and the extracranial regions of the head principally get their blood supply from the branches of the external carotid artery: superior thyroid artery, lingual artery, facial artery, occipital artery, posterior auricular artery, ascending pharyngeal artery, maxillary artery, and superficial temporal artery [1]. These branches undergo relatively frequent anatomical variations, and while most of them are clinically insignificant, they may alter surgical techniques or affect the outcome of clinical imaging studies related to certain diseases [2]. A successful preoperative radiological evaluation relies on prior knowledge of anatomical variations in the different branches of the external carotid artery. This fundamental skill can alert the vascular surgeon to the presence of unusual vessels and facilitate surgical intervention. For example, significant structural differences in the pattern of carotid artery bifurcation could potentially predispose an individual to plaque formation in the inner lining of the artery, requiring intervention for stroke prevention [3]. Additionally, ligating vessels during surgical emergencies, cosmetic surgeries, radical neck resections, visceral neck organ manipulation, and arterial angiograms before the operative procedure all require a deep and accurate understanding of the detailed anatomy of the superior thyroid, lingual, and facial artery branching. This knowledge is essential for better tracing the precise location and potential spread of malignancies, as improper ligation can result in serious consequences [4]. This is an instructive case report of a rare occurrence of a unilateral thyrolinguofacial trunk discovered during the dissection of a male African cadaver.

## Case presentation

**Figure 1:**
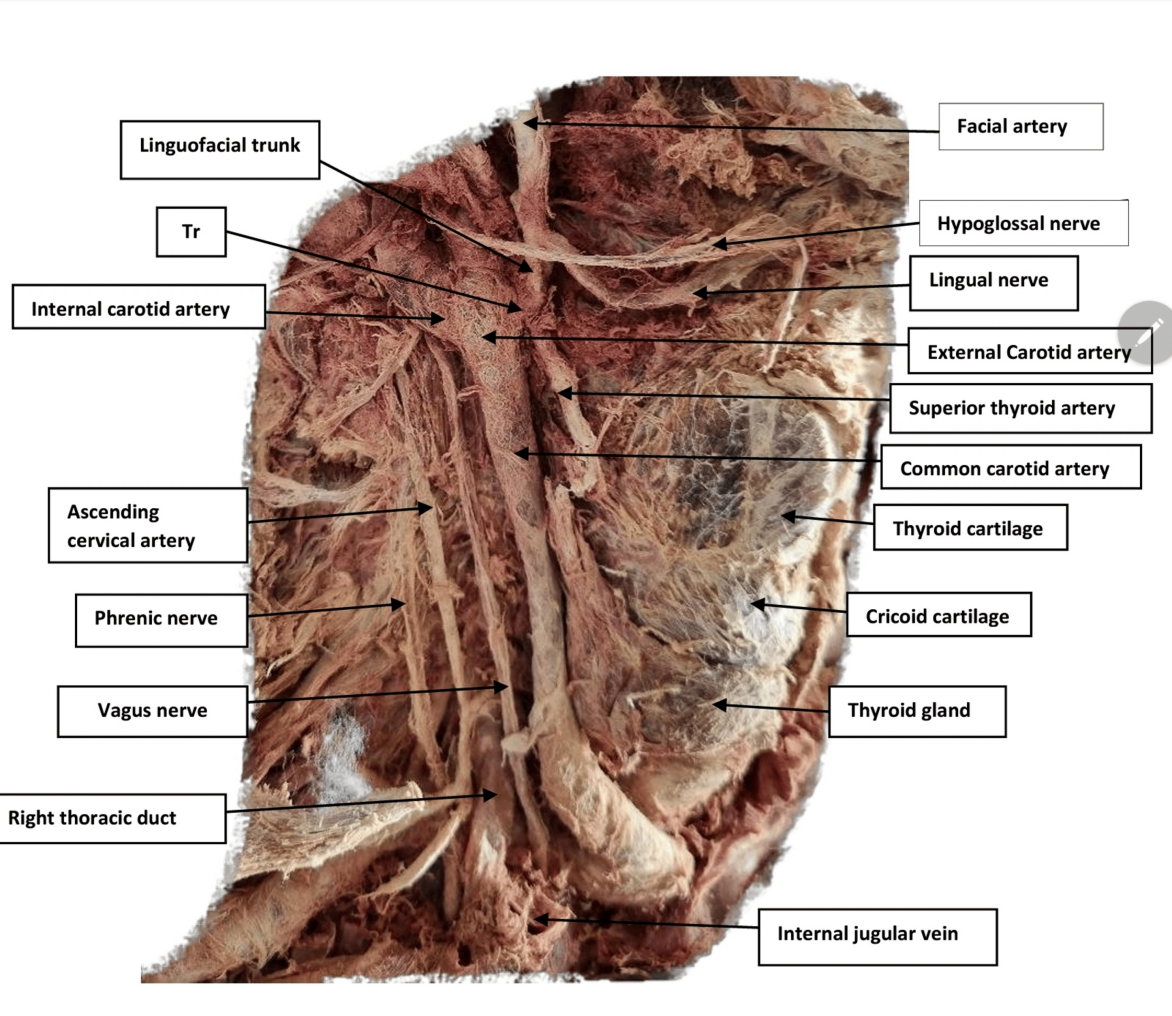
Photograph of the right side of the neck shows the course of the common carotid artery Tr, thyrolinguofacial trunk

**Figure 2:**
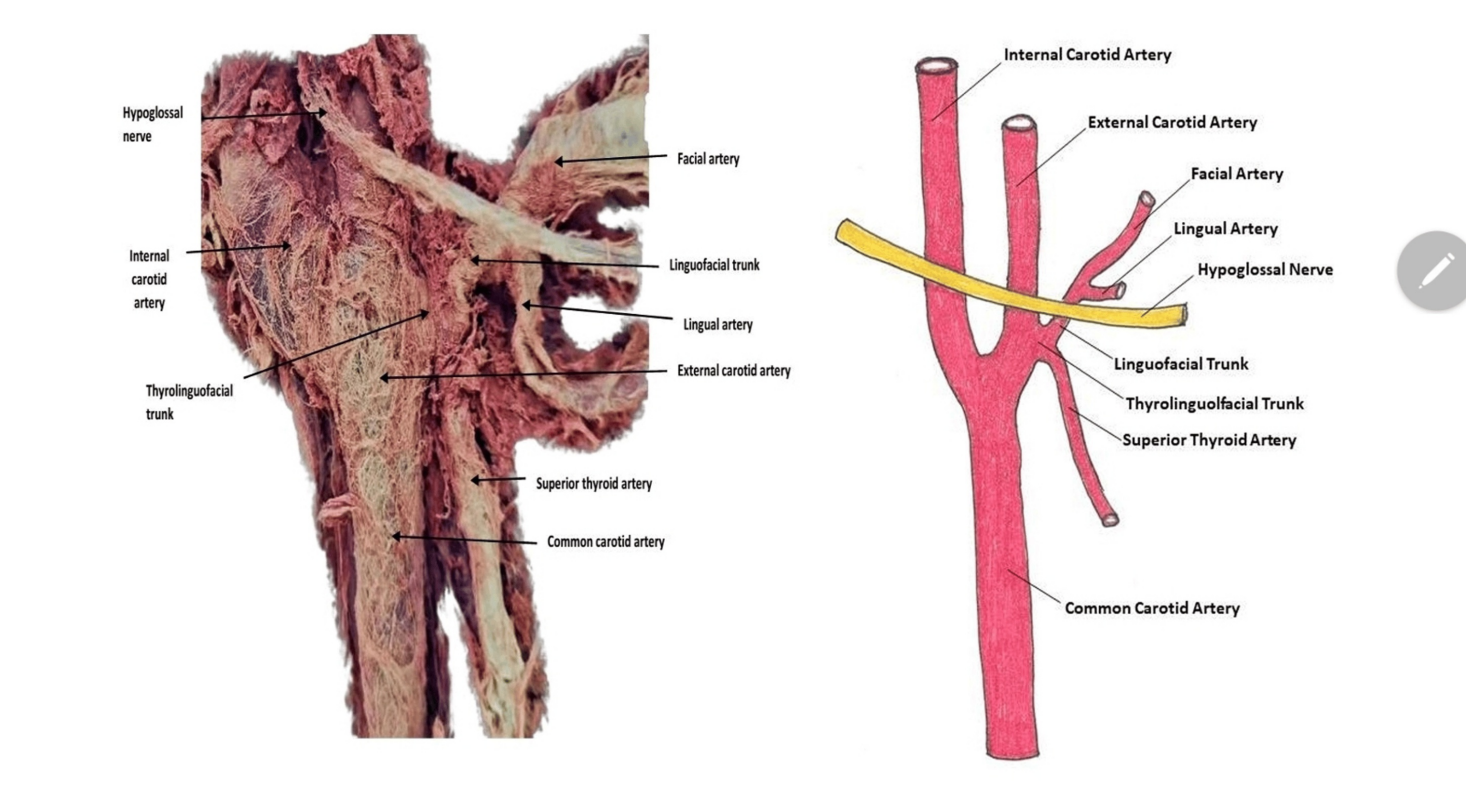
Ventral branches of external carotid artery showing thyrolinguofacial trunk The composite image on the left shows the bifurcation of the common carotid artery into the external carotid artery and internal carotid artery at the level of the hypoglossal nerve. The illustration on the right was created by the senior author (Peter J. Ward) of this study.

During the dissection of a male cadaver of African origin at the Anatomy Laboratory, Faculty of Medicine, Uzima University, Kisumu, Kenya, anatomical variations in the branches of the right external carotid artery were encountered. The initial dissection was conducted by students for hands-on experience, while a more detailed investigation was carried out by the author. The skin and subcutaneous tissues of the neck were lifted prior to dissecting the surrounding musculature. Superficial veins were identified, and their position in relation to neighboring structures was studied. Following the removal of the deep cervical fascia and underlying muscles, the right internal jugular vein and its tributaries were reflected to observe the common carotid artery. The common carotid artery is bifurcated into the internal carotid artery and external carotid artery at the superior margin of the thyroid cartilage, corresponding to the third cervical vertebra. We found an unexpected arterial trunk, the thyrolinguofacial trunk, on the right side, originating from the ventral part of the external carotid artery above the carotid bifurcation (Figure 1). Due to the fixed nature of the cadaver and the limited dissection tools, the details of the ventral branches of the external carotid artery were only explored in the neck and parotid regions. Measurements were taken using a caliper before the right unilateral variant was photographed. The thyrolinguofacial trunk in the cadaveric specimen under examination was the first branch to emerge from the ventral aspect of the external carotid artery, 3.8 mm above the carotid bifurcation. The length of the thyrolinguofacial trunk was 2.4 mm. After giving off the superior thyroid artery, it proceeded to course upward, anteriorly, and medially as the linguofacial trunk (Figure 2). The linguofacial trunk progressed for 9 mm before terminating into the lingual artery and facial artery above the greater horn of the hyoid bone in the submandibular region. During its course, the linguofacial trunk was crossed superficially by the hypoglossal nerve. The tortuous facial artery followed its normal course through the submandibular region onto the face, while the lingual artery looped inferomedially to enter the space below the tongue, supplying the tongue and the floor of the mouth.

## Discussion

Anomalies arising from variant anatomy generally do not interfere with the function of the human body and do not typically manifest as pathological nosological units. Under certain conditions, however, these abnormalities can worsen existing pathological states or even evoke new ones [5]. The cardiovascular system is the most variable part of the human body with the great vessels of the head and neck exhibiting the highest frequency of anomalies [6]. Accurate knowledge of vascular patterns will minimize the potential risk of iatrogenic bleeding during surgical procedures. The majority of arterial blood to the head and neck region originates from the common carotid artery, which bifurcates into the internal and external carotid arteries. These two arteries send out branches that perfuse the head and neck region and the intracranial structures. Morphological fluctuations in the origin, course, and distribution of those branches are not uncommon.

Various patterns of branching of the external carotid artery have been reported in the literature [7,8]. Based on a study by Ozgur (2008) [9] on the origin locations of the superior thyroid artery, lingual artery, and facial artery, in 90% of cases, these arteries originated as separate branches; linguofacial trunk cases accounted for 7.5%, and thyrolinguofacial trunk cases for 2.5%. Another study by Zümre [10] reported an incidence of the linguofacial trunk in 20% of cases, the thyrolinguofacial trunk in 2.5%, the thyrolingual trunk in 2.5%, and the occipitoauricular trunk in 12%. The thyrolinguofacial trunk is a rare occurrence, typically observed on one side of the body [10,11]. However, in a study by Monica et al. (2018), it was observed bilaterally [12].

The complex developmental pattern of the external carotid artery predisposes it to variations in its branching pattern. Due to insufficient knowledge about its development, the thyrolinguofacial trunk is rarely noticed. It is unknown if this pattern results initially from an anomaly of the external carotid artery, facial artery, superior thyroid artery, or even the lingual artery. Articles also predict that such arterial trunks could have been a result of remnants of the second aortic arch in the fetus. None of these can definitively determine its embryological origin; however, its existence proves critical in certain operations [12].

Imaging techniques such as computed tomography and magnetic resonance imaging have since replaced dissection in providing comprehensive visualization of blood vessels. Angiography is a less invasive procedure that saves time and offers more accurate and detailed visualization, particularly for the analytical description of both common and rare variations [13]. Therefore, angiography is the recommended tool for the successful characterization of arterial anatomical variations in the cervical area.

## Conclusions

Anatomists and clinicians working in the neck must have a detailed knowledge of normal anatomy and be able to recognize variations that may be encountered during various interventions and procedures. For example, surgeries such as carotid endarterectomy require exposure of the common carotid artery and many of its branches up to the level of the hypoglossal nerve. Variations in branching may restrict the surgeon’s field of view or create intraoperative challenges. This is just one example of a procedure in which complications related to the external carotid artery or its branches could arise, especially when an unanticipated variation in branching complicates the procedure. In addition to hemorrhage, damage to branches of the external carotid artery could lead to extensive tissue ischemia. Such negative outcomes can be prevented with a thorough understanding of variations like the thyrolinguofacial trunk.
